# Occupations Suitable for Women

**Published:** 1881-05

**Authors:** 


OCCUPATIONS SUITABLE FOR WOMEN.—We called attention editorially last year, and endeavored to point out some occupations that were filled by, and also others which are in every way suitable for females, but which are generally in the hands of males. In making our list we failed to think of another one, for which the female intelligence, taste and delicacy of hand are peculiarly well adapted, viz.: What is learned in SCHOOLS OF DESIGN. We copy below from the Daily *Gazette* of this city some appropriate editorial remarks upon the subject:

"As to the humanizing, refining, ennobling nature of this species of labor it is impossible to say too much. The beautiful is always the appropriate, the true. It is sought for and learned only in the study of nature and her ever-wondrous, ever-changing forms and colors. All the delightful and profitable employments that are fostered and developed in these Schools of Design are not now merely open to women, but they are preferred in them. And they should furnish, especially to those who have been born to an expectation of freedom from labor, which has been disappointed, a means of livelihood, congenial, lady-like and remunerative. In view of the possible monetary calamities which may come sooner or later to those who expect them the least, would it not be well for all who are blessed with that species of talent, and have now the means and opportunities to make it useful for future emergency, to cultivate it with a special view to its use in case of misfortune? The tender and delicate girl who now rides in her carriage, and is wrapped in every appliance of luxury, in a few short years, perhaps months, may find herself thrown upon a cold world, amid a crowd of similar work-seekers, asking for daily support. No parent in this country, where wealth is so precarious and its duration so impossible to insure, can be said to perform his duty to his daughters if he neglects to place them as nearly as possible upon an equal footing with his sons, and provide them with the only species of property which can be made to outlast revulsion and depreciation—the capability of earning their own living."

